# Low‐load resistance exercise with and without blood flow restriction: Which is more effective for increasing local muscle endurance and why?

**DOI:** 10.1113/EP091872

**Published:** 2024-03-23

**Authors:** Mathias Wernbom

**Affiliations:** ^1^ Department of Health and Rehabilitation, Institute of Neuroscience and Physiology Sahlgrenska Academy University of Gothenburg Gothenburg Sweden

**Keywords:** capillarization, hypoxia, ischaemia, mitochondria, muscle hypertrophy, occlusion, vasodilatation

1

The first studies which demonstrated increased muscle strength and size after low‐load resistance exercise combined with blood flow restriction (BFR) were published about 25 years ago. Ever since the first studies, BFR resistance exercise (BFR‐RE) has continued to attract great interest. However, in an early review on BFR‐RE (Wernbom et al., 2008), it was remarked that any unique effects of cuff occlusion per se during exercise had yet to be determined because the increased training effects with low‐load BFR‐RE over work‐matched low‐load resistance exercise without BFR observed in the studies published up to that date might simply have been due to greater effort in the BFR‐RE condition. In the same review (Wernbom et al., 2008), we also proposed that training at loads as low as 20%–30% of one repetition maximum (1RM) in certain exercises could induce muscle hypertrophy even without BFR if the training was taken to voluntary failure. Some years later, Farup et al. (2015) showed similar muscle hypertrophy after low‐load dynamic elbow flexion training to failure with and without BFR. However, Farup et al. (2015) did not investigate the effects of the two regimes on local muscle endurance at submaximal loads.

In this issue of *Experimental Physiology*, Ida and Sasaki (2024) report that 6 weeks of low‐load (30% of 1RM) dynamic elbow flexion exercise to failure with free blood flow (FBF) resulted in greater gains in dynamic muscle endurance compared with BFR‐RE to failure at the same load, as assessed by the number of repetitions performed at 30% of 1RM. Furthermore, they report that the increases in local muscle endurance were correlated with the exercise volume over the 6 weeks of training, suggesting that BFR may attenuate improvements in muscle endurance adaptations to low‐load resistance exercise by decreasing the accumulated training volume. In contrast, the two regimes resulted in similar gains in muscle thickness and strength, thus largely corroborating the results of Farup et al. (2015). There were also no significant differences in the improvements in the time required for maximal voluntary isometric contraction (MVC) force to drop to 50% (half‐time) during repeated intermittent MVCs, although the half‐time increases appeared to be more robust in the BFR‐trained arm. In the absence of significant differences in whole muscle size and strength increases, other adaptations must have contributed to the differences in the local muscle endurance tests.

Ida and Sasaki (2024) speculate that BFR may have impaired mitochondrial adaptations by decreasing the reliance on aerobic metabolism during exercise over the training period. They base this proposal on recent findings from the group of Jamie Burr and colleagues that BFR attenuated both reactive oxygen species (ROS) emissions from mitochondria (Petrick et al., 2019) and the improvement in mitochondrial respiratory capacity in the vastus lateralis muscle following 6 weeks of one‐leg squat training (Pignanelli et al., 2020). Notably, Petrick et al. (2019) also reported that reduced O_2_ tension reduced emission of ROS (determined by release of H_2_O_2_) in human permeabilized muscle fibres in vitro. Lower mitochondrial release of ROS may then in turn lead to less signals for muscle endurance adaptations. The muscle tissue oxygenation results in Experiment 1 in Ida and Sasaki (2024) support the possibility of lower oxygen tension in the BFR‐trained arm during training.

Support for the scenario of impaired mitochondrial adaptations due to low O_2_ tension during exercise can also be found in a study by Bakkman et al. (2007), who investigated adaptations to one‐legged cycle training during hypoxia and normoxia at the same relative intensity (65% of the maximal power output in hypoxia and normoxia, respectively). Bakkman et al. (2007) reported that citrate synthase activity increased significantly after training during normoxic conditions (+20.8%) but remained unchanged after hypoxia training (+4.5%, non‐significant increase) with a significant difference between conditions. Furthermore, the maximal ADP‐stimulated respiration expressed per weight of muscle tended to increase after normoxic training (+31.2%, *P* < 0.08) but not after hypoxic training (+3.2%). The picture that emerges from these studies is that local hypoxia may attenuate exercise‐induced muscle mitochondrial adaptations compared with more normoxic conditions.

On the other hand, a study from the group of Kristian Vissing and coworkers (Groennebaek et al., 2018) demonstrated marked increases in mitochondrial respiratory function and mitochondrial protein synthesis rates after 6 weeks of training with multiple sets of low‐load BFR‐RE. Although speculative, the seemingly discrepant findings between the studies of Groennebaek et al. (2018) and Pignanelli et al. (2020) with regard to improvements in mitochondrial function may in part have been due to differences in the BFR pressures employed during training. Groennebaek et al. (2018) used a 14 cm cuff inflated to 50% of arterial occlusion pressure (AOP) in a supine position (corresponding to ∼40% of AOP in a seated position), on average 79 mm Hg of BFR pressure. Pignanelli et al. (2020) used an 11 cm cuff inflated to 60%–70% of AOP in a seated position, on average ∼155 mm Hg of BFR pressure. These differences in relative BFR pressures may well result in different muscle tissue oxygenation levels during exercise, especially in the rest periods between sets. In contrast, low‐load resistance exercise to failure without BFR and BFR‐RE to failure resulted in similar increases in capillary content (Pignanelli et al., 2020). Increased capillarization and/or improved vasodilatation as well as other mechanisms could perhaps to a certain extent compensate for any suboptimal mitochondrial adaptations that might result from severe hypoxia induced by high pressure BFR‐RE.

In any case, the discrepant findings between different studies make it readily apparent that a lot more research is still needed to shed more light on how BFR‐RE impacts the mechanisms underpinning improved muscle endurance, and how the training volume as well as the relative BFR pressures and the mode of BFR (intermittent vs. continuous) influence each of these mechanisms. Meanwhile, the results of Ida and Sasaki (2024) also serve to remind us that fatiguing low‐load resistance exercise without externally applied BFR can be a simple yet remarkably effective mode of training for increasing local muscle endurance capacity, in addition to inducing significant increases in muscle strength and size.

## AUTHOR CONTRIBUTIONS

Sole author.

## CONFLICT OF INTEREST

No competing interests declared.

## FUNDING INFORMATION

No funding was used for this article.
